# Respiratory Syncytial Virus Vaccination in Solid Organ Transplant Recipients: Interim Findings From a Phase 3 Trial of mRNA-1345

**DOI:** 10.1093/cid/ciag108

**Published:** 2026-02-18

**Authors:** Erick F Mayer, Ann R Falsey, Cameron R Wolfe, Erica Herc, Fiona Burns, Dima Kabbani, Deepali Kumar, Anisha Mannan, Fahua She, Lan Lan, Shannon McGrath, Archana Kapoor, Frances Priddy

**Affiliations:** Moderna, Inc., Cambridge, Massachusetts, USA; University of Rochester School of Medicine, Rochester, NewYork, USA; Duke University Medical Center, Durham, North Carolina, USA; Henry Ford Hospital, Detroit, Michigan, USA; University College London & Royal Free London NHS Foundation Trust, London, United Kingdom; University of Alberta, Edmonton, Alberta, Canada; University Health Network, Toronto, Ontario, Canada; Moderna, Inc., Cambridge, Massachusetts, USA; Moderna, Inc., Cambridge, Massachusetts, USA; Moderna, Inc., Cambridge, Massachusetts, USA; Moderna, Inc., Cambridge, Massachusetts, USA; Moderna, Inc., Cambridge, Massachusetts, USA; Moderna, Inc., Cambridge, Massachusetts, USA

**Keywords:** respiratory syncytial virus, safety, immunogenicity, mRNA RSV vaccine, solid organ transplant

## Abstract

**Background:**

Solid organ transplant (SOT) recipients are at increased risk for severe respiratory syncytial virus (RSV) disease due to chronic immunosuppression.

**Methods:**

In this ongoing, open-label, phase 3 trial, adults ≥18 years with a history of liver, kidney, or lung transplant received 2 doses of mRNA-1345 RSV vaccine (50 µg) 56 days apart. Primary endpoints were tolerability, safety, and RSV-A/RSV-B neutralizing antibody (nAb) responses on Day 85; secondary endpoints included immunogenicity on Days 29 and 181. Cell-mediated immunity was an exploratory endpoint assessed in a subset of participants.

**Results:**

146/150 participants (median: age, 57 years; time since transplant, 4.7 years) received both doses. Reactogenicity was mild to moderate and transient. No vaccine-related discontinuations, deaths, adverse events of special interest (AESIs), or events of transplant rejection were reported within 28 days after any dose. One dose was immunogenic across all SOT types, with 4.9- and 3.4-fold increases in RSV-A/RSV-B nAb GMTs by Day 29, respectively. A second dose resulted in modest additional increases over baseline (RSV-A, 7.1-fold; RSV-B, 5.2-fold). Added benefit of the second dose was more apparent in participants with kidney and lung transplant, <2 years post-transplant, and on mycophenolate. Responses remained above baseline through Day 181. Polyfunctional CD4⁺ T-cell responses were robust and sustained; CD8⁺ responses were also observed.

**Conclusions:**

mRNA-1345 was well tolerated and immunogenic in SOT recipients. A single dose induced nAb responses across subgroups, with potential additional benefit from a second dose in specific groups. Durable antibody and cellular responses support mRNA-1345 as a preventive strategy for RSV in this vulnerable population.

**Clinical Trials Registration:**

NCT06067230.

## Teaser

mRNA-1345 RSV vaccine was well tolerated, with no safety concerns. A single dose was immunogenic across all SOT subgroups; select participants—such as kidney or lung recipients, those <2 years post-transplant, or on mycophenolate—may benefit from a second dose.

Respiratory syncytial virus (RSV) is a leading cause of acute respiratory illness globally, with particularly high morbidity and mortality among infants, older adults, and individuals with chronic conditions [1–3]. Solid organ transplant (SOT) recipients are especially vulnerable to RSV-associated lower respiratory tract disease (LRTD) due to chronic immunosuppression, with consequences that include hospitalization, secondary infections, allograft dysfunction, and increased mortality [4–7]. Among lung transplant recipients (LTRs), RSV is relatively common and can lead to serious complications, including progression to LRTD and chronic lung allograft dysfunction [4, 5, 8].

Despite recent approvals of adult RSV vaccines, immunocompromised populations, which include SOT recipients, remain underrepresented in clinical trials. Observational data among adults ≥ 60 years suggest substantial vaccine effectiveness in immunocompromised groups, including SOT recipients, but subgroup-specific estimates remain imprecise, and prospective data in SOT recipients are limited [9]. In 1 phase 3 study of an unadjuvanted prefusion (preF) RSV vaccine in immunocompromised or renally impaired adults, 37% of whom were SOT recipients, 2 doses 1 month apart were well tolerated and elicited measurable neutralizing antibody (nAb) responses [10]. However, more data are needed to fully optimize effective vaccination strategies in this vulnerable population.

mRNA-1345 is a lipid nanoparticle encapsulated mRNA-based vaccine encoding the preF-stabilized RSV F-protein. The vaccine is approved in the United States for prevention of RSV-LRTD in adults aged ≥60 years and in adults aged 18–59 years at increased risk; in other regions, approval currently applies to adults aged ≥60 years [11–13]. In older adults, a single 50-µg dose elicits robust nAb responses and demonstrates protective efficacy against RSV-A and RSV-B [14, 15]. Immunogenicity has likewise been demonstrated in adults aged 18–59 years with high-risk medical conditions, where nAb responses met prespecified criteria for noninferiority to those observed in adults aged ≥60 years [16].

This study is part of a larger phase 3 trial evaluating the safety and immunogenicity of mRNA-1345 in adults at increased risk of RSV-LRTD. Because immunocompromised patients may have attenuated vaccine responses, and prior experience with SARS-CoV-2 mRNA vaccines in SOT recipients has shown that additional doses can enhance immunogenicity [17], this part of the study was designed to evaluate 2 doses given 56 days apart, with prespecified immunologic assessments after the first dose. Here, we report interim findings in SOT recipients (kidney, liver, lung) through Day 271.

## MATERIALS AND METHODS

### Study Design and Participants

This ongoing, open-label, phase 3 study (NCT06067230) evaluates the safety, tolerability, and immunogenicity of mRNA-1345 in adult SOT recipients. Participants were enrolled at 22 centers (16 in the United States, 2 in Canada, and 4 in the United Kingdom). Eligible participants were ≥18 years of age; had received a kidney, liver, or lung transplant ≥180 days before enrollment; and were receiving maintenance immunosuppressive therapy. Enrollment targeted approximately equal distribution (∼33%) across the 3 transplant types. Individuals with unstable medical conditions or prior receipt of an RSV vaccine were excluded. Full eligibility criteria are provided in the Supplementary Data. The trial is planned to continue for 24 months after the first dose; this report includes interim data through a median of approximately 9 months of follow-up.

The study was conducted in accordance with the principles of the Declaration of Helsinki and International Council for Harmonisation Good Clinical Practice (ICH GCP) guidelines. The protocol was approved by all applicable institutional review boards, and all participants provided written informed consent prior to enrollment.

### Objectives

The primary objectives were to evaluate safety and tolerability and to assess immunogenicity as measured by geometric mean titers (GMTs) of RSV-A and RSV-B nAbs on Day 85 following 2 doses administered 56 days apart. Secondary objectives included evaluation of GMTs on Days 29 and 181; seroresponse rates (SRRs, ≥4-fold rise from baseline) on Days 29, 85, and 181; and geometric mean fold rise (GMFR) from baseline at each time point, as well as binding antibody responses to RSV preF protein. All immunogenicity analyses were conducted by SOT type (lung, liver, kidney). Assessment of RSV-specific cell-mediated immunity (CMI) in a subset of participants was assessed as an exploratory objective.

### Study Interventions and Procedures

Participants received 2 doses of mRNA-1345 (50 μg) intramuscularly on Days 1 and 57.

Solicited local and systemic adverse reactions (ARs) were collected via e-diary for 7 days after each injection. Unsolicited adverse events (AEs) were collected for 28 days following each injection. Medically attended AEs (MAAEs), defined as AEs leading to an unscheduled visit to a healthcare provider, were recorded through Day 271, and AEs of special interest (AESIs), serious AEs (SAEs), and AEs leading to study discontinuation were collected through the end of the study.

Serum samples were collected at baseline and on Days 29, 57, 85, and 181 for immunogenicity assessment. nAbs to RSV-A and RSV-B were measured using validated microneutralization assays. Respiratory syncytial virus preF binding antibody concentrations were assessed using validated Luminex-based immunoassay [18].

Respiratory syncytial virus disease surveillance included active symptom monitoring during the RSV season and symptom-triggered illness visits with RT-PCR testing when prespecified criteria were met. Respiratory syncytial virus disease endpoints were exploratory and are not reported in this manuscript.

A subset of participants from sites equipped for peripheral blood mononuclear cell (PBMC) processing participated in CMI assessments. Peripheral blood mononuclear cells were collected at predefined time points (Days 1, 15, 57, 71, and 181). RSV-F–specific T-cell responses were assessed by intracellular cytokine staining upon ex vivo stimulation with an RSV-F peptide pool. CD4⁺ and CD8⁺ T cells were evaluated for expression of type 1 and type 2 cytokines, as well as additional functional markers, including CD40 ligand (CD40L). Additional information is provided in the Supplementary Data.

### Statistical Analysis

The per-protocol (PP) set was used for immunogenicity analyses and included participants who received both doses, had baseline and ≥1 post-dose assessment, and had no major protocol deviations that impact immune response. The safety set included all participants who received ≥1 dose. All analyses were descriptive; GMTs, GMFRs, and SRRs were summarized with 95% confidence intervals (CIs). The 95% CIs of GMTs were calculated based on the *t* distribution of the log-transformed values and then back-transformed to the original scale. The number and percentage of participants with seroresponse at each time point were provided with 2-sided 95% CIs using the Clopper–Pearson method. Subgroup analyses were conducted by transplant type, time since transplant, and concomitant immunosuppressive regimen. All analyses used SAS software version 9.4 (SAS Institute, Cary, North Carolina). Additional information is provided in the Supplementary Data.

## RESULTS

### Participants

**Figure 1.**
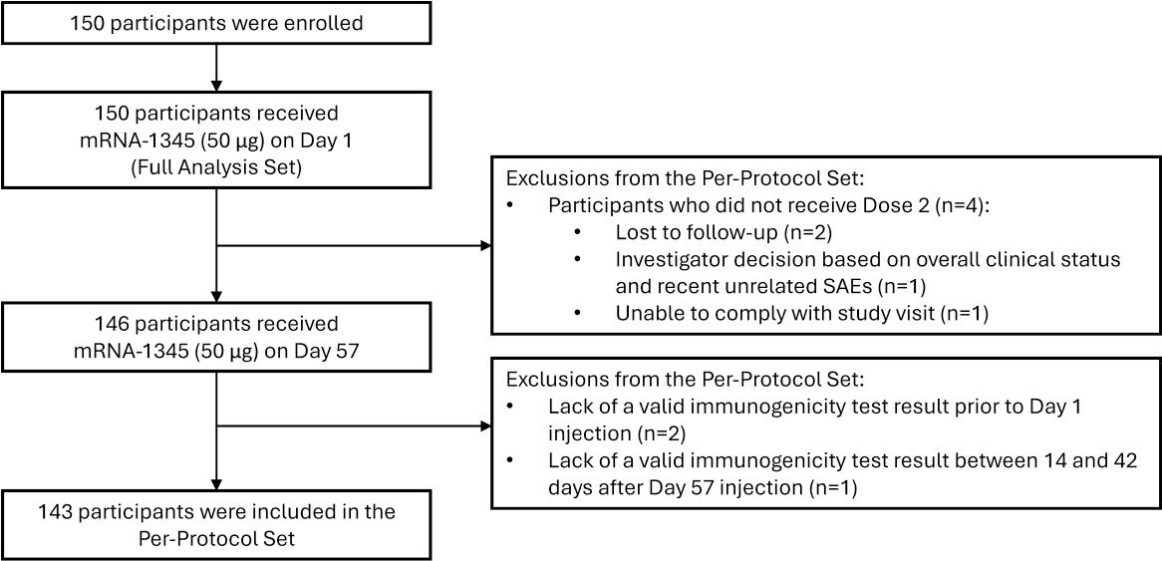
Participant flow diagram. All 150 participants received mRNA-1345 on Day 1 (safety set). Of these participants, 146 received the Day 57 dose; 143 participants were included in the per-protocol set based on valid immunogenicity test results. Abbreviation: SAE, serious adverse event.

A total of 150 adult participants were enrolled between October 2023 and July 2024. All 150 participants received Dose 1, and 146 (97.3%) received Dose 2 (Figure 1). The median age was 57 years (range, 24–80), 37.3% were female, and 70.0% were White. Transplant types included kidney (32.0%), liver (32.0%), lung (30.7%), and multiple organs (5.3%). The median time since transplant to enrollment was 4.7 years (range, 0.5–29.8), with 26.7% within 2 years of transplantation. The most common immunosuppressive regimens included tacrolimus, mycophenolate, and steroids (45.3%), tacrolimus alone (18.0%), and tacrolimus with mycophenolate (16.7%). Additional demographic and clinical details are provided in Table 1.

**Table 1.** Baseline Demographics of Study Participants (Safety Set)

	mRNA-1345 50 µg (N = 150)
Age, y	
Mean (SD)	55.1 (12.45)
Median (min, max)	57.0 (24, 80)
Age group, n (%)	
18–49 y	45 (30.0)
≥50 y	105 (70.0)
Sex, n (%)	
Male	94 (62.7)
Female	56 (37.3)
Race group, n (%)^a^	
White	105 (70.0)
Black	34 (22.7)
Asian	2 (1.3)
Other	4 (2.7)
Missing	5 (3.3)
Ethnicity, n (%)	
Hispanic or Latino	15 (10.0)
Not Hispanic or Latino	134 (89.3)
Solid organ transplant, n (%)^b^	
Kidney	48 (32.0)
Liver	48 (32.0)
Lung	46 (30.7)
Multiple	8 (5.3)
Time since transplantation, years^c^	
Mean (SD)	7.05 (6.588)
Median (min, max)	4.73 (0.5, 29.8)
Concomitant immunosuppressive therapies, n (%)^d^	
Antiproliferative agents	106 (70.7)
Calcineurin inhibitors	145 (96.7)
mTOR inhibitors	7 (4.7)
Steroids	91 (60.7)
Other	4 (2.7)
None^e^	2 (1.3)
Combination of concomitant immunosuppressive therapies, n (%)^f^	
Mycophenolate, tacrolimus	25 (16.7)
Mycophenolate, tacrolimus, steroids	68 (45.3)
Tacrolimus alone	27 (18.0)
Other combinations	28 (18.7)
None^e^	2 (1.3)

Percentages were based on the number of participants in the safety set.

Abbreviations: eCRF, electronic case report form; IST, immunosuppressive therapy; mTOR, mechanistic target of rapamycin.

^a^Other race included American Indian or Alaska Native, Native Hawaiian or other Pacific Islander, other, or multiple.

^b^Derived from data collected on Targeted Medical History and General Medical History eCRFs.

^c^If a participant had undergone multiple transplants, only the most recent transplant was included in the summary.

^d^IST types included (a) antiproliferative agents: azathioprine and mycophenolate; (b) calcineurin inhibitors: tacrolimus and cyclosporine; (c) mTOR inhibitors: everolimus and sirolimus; (d) steroids: dexamethasone, fludrocortisone, hydrocortisone, methylprednisolone, prednisone, and prednisolone; and (e) other: antithymocyte immunoglobulin, basiliximab, belatacept, and vedolizumab.

^e^Two participants were initially recorded as not receiving concomitant immunosuppressive therapy due to missing entries in the electronic data capture system. Source documentation confirmed that both participants were receiving mycophenolate, tacrolimus, and steroids.

^f^Concomitant combinations included tacrolimus, mycophenolate, and steroids; tacrolimus and mycophenolate; tacrolimus; and other rare combinations.

### Safety

**Figure 2.**
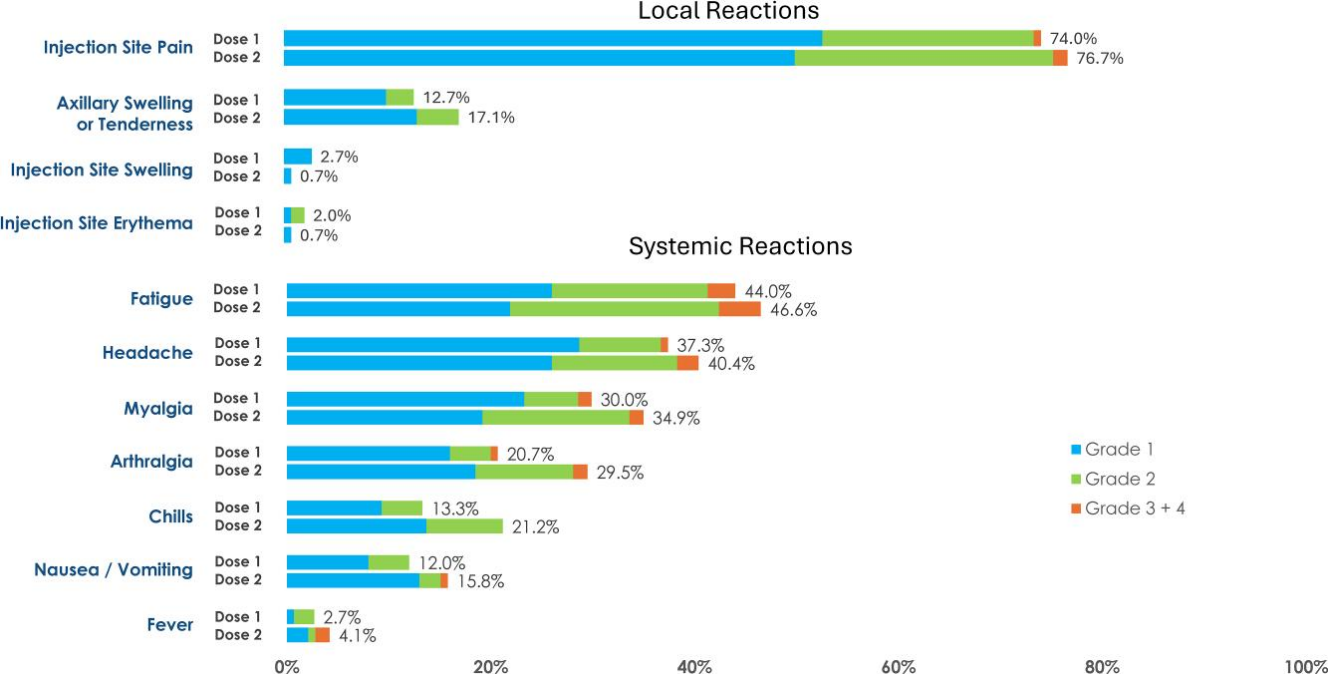
Solicited adverse reactions within 7 d after Dose 1 and Dose 2 of mRNA-1345 (50 μg) in solid organ transplant recipients (solicited safety set). Dose 1, n = 150; Dose 2, n = 146. Percentages were based on the number of exposed participants who submitted any data for the event. One grade 4 systemic reaction of arthralgia was reported for 1 participant (0.7%) after Dose 2.

Solicited local ARs occurred in 74.0% of participants after Dose 1 and 77.4% after Dose 2, while systemic ARs were reported in 64.0% and 64.4% of participants, respectively (Supplementary Table 1). Most solicited local and systemic ARs occurred within 1–2 days after injection (Figure 2). The median duration was 2 days for solicited local ARs and 2.5–3 days for solicited systemic ARs (Supplementary Table 2). Injection site pain was the most frequent local AR, and fatigue, headache, myalgia, and arthralgia were the most frequent systemic ARs.

One participant with a bilateral lung transplant experienced a grade 4 event of arthralgia 6 days after Dose 2. The event was assessed by the investigator as related to study vaccination and was also classified as an SAE and MAAE. Two weeks prior to the event of arthralgia, the participant received rabbit anti-thymocyte globulin for treatment of preexisting transplant rejection, and the episode of arthralgia was treated with intravenous methylprednisolone, followed by prednisone taper for possible serum sickness attributed to rabbit anti-thymocyte globulin therapy, resolving after 2 days.

Within 28 days of any dose, unsolicited AEs and MAAEs occurred in 34.0% and 24.7% of participants, respectively (Supplementary Table 3). Injection-related unsolicited AEs were reported in 3 participants (2.0%). The previously described SAE of arthralgia was the only MAAE assessed as related by the investigator. No deaths, AEs leading to study discontinuation, or AESIs were reported in the 28-day post-dose window.

As of the data cutoff (21 April 2025), SAEs were reported in 28.7% of participants and MAAEs were reported in 65.3% of participants (Supplementary Table 4). There were no additional SAEs and no AESIs assessed as related by the investigator. Biopsy-confirmed organ rejection was reported in 5 participants, including 2 participants with a medical history of organ rejection; all cases were considered not related by the investigator. Four of these cases occurred in LTRs and 1 in a kidney transplant recipient.

One fatal event occurred in a LTR with multiple comorbidities. The participant was hospitalized on Day 225 for cytomegalovirus colitis, subsequently developed multiple complications, and later died of worsening preexisting chronic kidney disease (Day 287). All AEs during the hospitalization, including the fatal event, were assessed by the investigator as not related to study vaccination.

No events of anaphylaxis, Guillain–Barré syndrome, acute disseminated encephalomyelitis, Bell's palsy, or acute myocarditis/pericarditis were reported.

### Immunogenicity

A single dose of mRNA-1345 was immunogenic across all SOT types. By Day 29, RSV-A nAbs GMTs increased to 9381.18 IU/mL (95% CI, 7333.39–12 000.79), with a GMFR of 4.9 (95% CI, 3.87–6.25), and RSV-B nAbs GMT increased to 2838.18 IU/mL (95% CI, 2283.77–3527.17), with a GMFR of 3.4 (95% CI, 2.82–4.05) (Table 2).

**Table 2.** Summary of RSV-A and RSV-B Neutralizing Antibody GMTs and GMFRs After mRNA-1345 Vaccine Administered on Day 1 and Day 57 in Adult Solid Organ Transplant Recipients by Visit up to Day 181 (Per-Protocol Set)

	mRNA-1345 50 µg (N = 143)
RSV-A neutralizing antibody titer (IU/mL)	
Baseline (Day 1)	
n^a^	143
GM titer, 95% CI^b^	1907.00 (1580.49, 2300.96)
Day 29	
n^a^	143
GM titer, 95% CI^b^	9381.18 (7333.39, 12000.79)
N1	143
GM fold rise, 95% CI^b^	4.92 (3.87, 6.25)
Seroresponse, n (%),^c,d^ 95% CI^e^	73 (51.0) (42.6, 59.5)
Day 57	
n^a^	142
GM titer, 95% CI^b^	9070.29 (7137.08, 11527.14)
N1	142
GM fold rise, 95% CI^b^	4.78 (3.78, 6.04)
Seroresponse, n (%),^c,d^ 95% CI^e^	75 (52.8) (44.3, 61.2)
Day 85	
n^a^	141
GM titer, 95% CI^b^	13 666.87 (10 811.92, 17275.70)
N1	141
GM fold rise, 95% CI^b^	7.07 (5.67, 8.80)
Seroresponse, n (%),^c,d^ 95% CI^e^	91 (64.5) (56.0, 72.4)
Day 181	
n^a^	138
GM titer, 95% CI^b^	8514.51 (6655.05, 10893.52)
N1	138
GM fold rise, 95% CI^b^	4.46 (3.59, 5.54)
Seroresponse, n (%),^c,d^ 95% CI^e^	73 (52.9) (44.2, 61.4)
RSV-B neutralizing antibody titer (IU/mL)	
Baseline (Day 1)	
n^a^	143
GM titer, 95% CI^b^	849.01 (724.42, 995.01)
Day 29	
n^a^	142
GM titer, 95% CI^b^	2838.18 (2283.77, 3527.17)
N1	142
GM fold rise, 95% CI^b^	3.38 (2.82, 4.05)
Seroresponse, n (%),^c,d^ 95% CI^e^	57 (40.1) (32.0, 48.7)
Day 57	
n^a^	142
GM titer, 95% CI^b^	2923.11 (2366.14, 3611.19)
N1	142
GM fold rise, 95% CI^b^	3.45 (2.89, 4.12)
Seroresponse, n (%),^c,d^ 95% CI^e^	55 (38.7) (30.7, 47.3)
Day 85	
n^a^	142
GM titer, 95% CI^b^	4427.18 (3594.82, 5452.28)
N1	142
GM fold rise, 95% CI^b^	5.17 (4.34, 6.17)
Seroresponse, n (%),^c,d^ 95% CI^e^	80 (56.3) (47.8, 64.6)
Day 181	
n^a^	138
GM titer, 95% CI^b^	3155.79 (2593.43, 3840.10)
N1	138
GM fold rise, 95% CI^b^	3.78 (3.21, 4.45)
Seroresponse, n (%),^c,d^ 95% CI^e^	63 (45.7) (37.2, 54.3)

N1 is the number of participants with non-missing data at baseline and the corresponding post-baseline time point. Antibody values reported as below the LLOQ were replaced by 0.5 × LLOQ. Values greater than the ULOQ were replaced by the ULOQ.

Abbreviations: CI, confidence interval; GM, geometric mean; LLOQ, lower limit of quantification; ULOQ, upper limit of quantification.

^a^Number of participants with non-missing data at the time point (baseline or post-baseline).

^b^95% CI was calculated based on the *t* distribution of the log-transformed values or the difference in the log-transformed values for GM value and GM fold rise, respectively, then back-transformed to the original scale for presentation.

^c^Seroresponse at a participant level was defined as a change from below the LLOQ to equal or above 4 × LLOQ, or at least a 4-fold increase if baseline was equal to or above the LLOQ.

^d^Number of participants who met the criterion at the time point. Percentages were based on N1.

^e^95% CI was calculated using the Clopper–Pearson method.

Following the second dose, additional increases over baseline were observed. At Day 85, GMTs were 13 666.87 (95% CI, 10 811.92–17 275.70) for RSV-A (GMFR, 7.1; 95% CI, 5.67–8.80) and 4427.18 (95% CI, 3594.82–5452.28) for RSV-B (GMFR, 5.2; 95% CI, 4.34–6.17).

nAb responses remained above baseline through Day 181, with GMFRs of 4.5 (95% CI, 3.59–5.54) for RSV-A and 3.8 (95% CI, 3.21–4.45) for RSV-B.

Seroresponse rates were 51.0% (95% CI, 42.6–59.5) for RSV-A and 40.1% (95% CI, 32.0–48.7) for RSV-B at Day 29 (Supplementary Table 5). Seroresponse rate increased following the second dose, reaching 64.5% (95% CI, 56.0–72.4) for RSV-A and 56.3% (95% CI, 47.8–64.6) for RSV-B at Day 85. At Day 181, SRRs remained above baseline at 52.9% (95% CI, 44.2–61.4) for RSV-A and 45.7% (95% CI, 37.2–54.3) for RSV-B.

**Figure 3.**
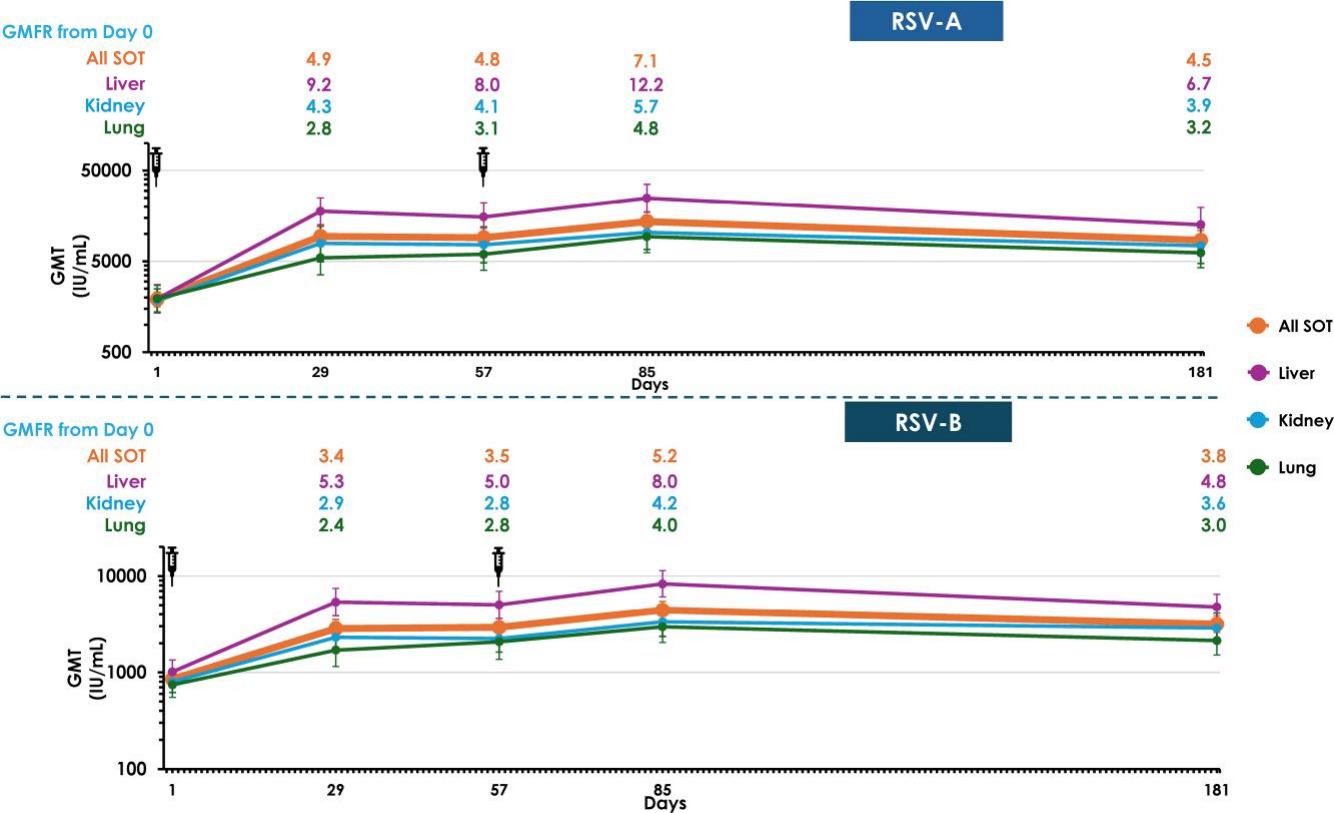
RSV-A and RSV-B neutralizing antibody GMTs in solid organ transplant recipients by transplant type and time point (per-protocol set). Antibody values reported as below the LLOQ were replaced by 0.5 × LLOQ. Values greater than the ULOQ were replaced by the ULOQ. Solid organ transplant type was derived from data collected via interactive response technology. Number of participants with non-missing data at the time point (baseline or post-baseline). 95% confidence intervals were calculated based on the *t* distribution of the log-transformed values or the difference in the log-transformed values for GMT value and GMFR, respectively, then back-transformed to the original scale for presentation. Abbreviations: GMFR, geometric mean fold rise; GMT, geometric mean titer; LLOQ, lower limit of quantification; RSV, respiratory syncytial virus; SOT, solid organ transplant; ULOQ, upper limit of quantification.

All transplant subgroups mounted nAb responses, with similar temporal patterns of increase. Liver transplant recipients had higher GMTs at all time points following injection, while kidney and lung recipients exhibited more pronounced increases between Dose 1 and Dose 2 (Figure 3).

**Figure 4.**
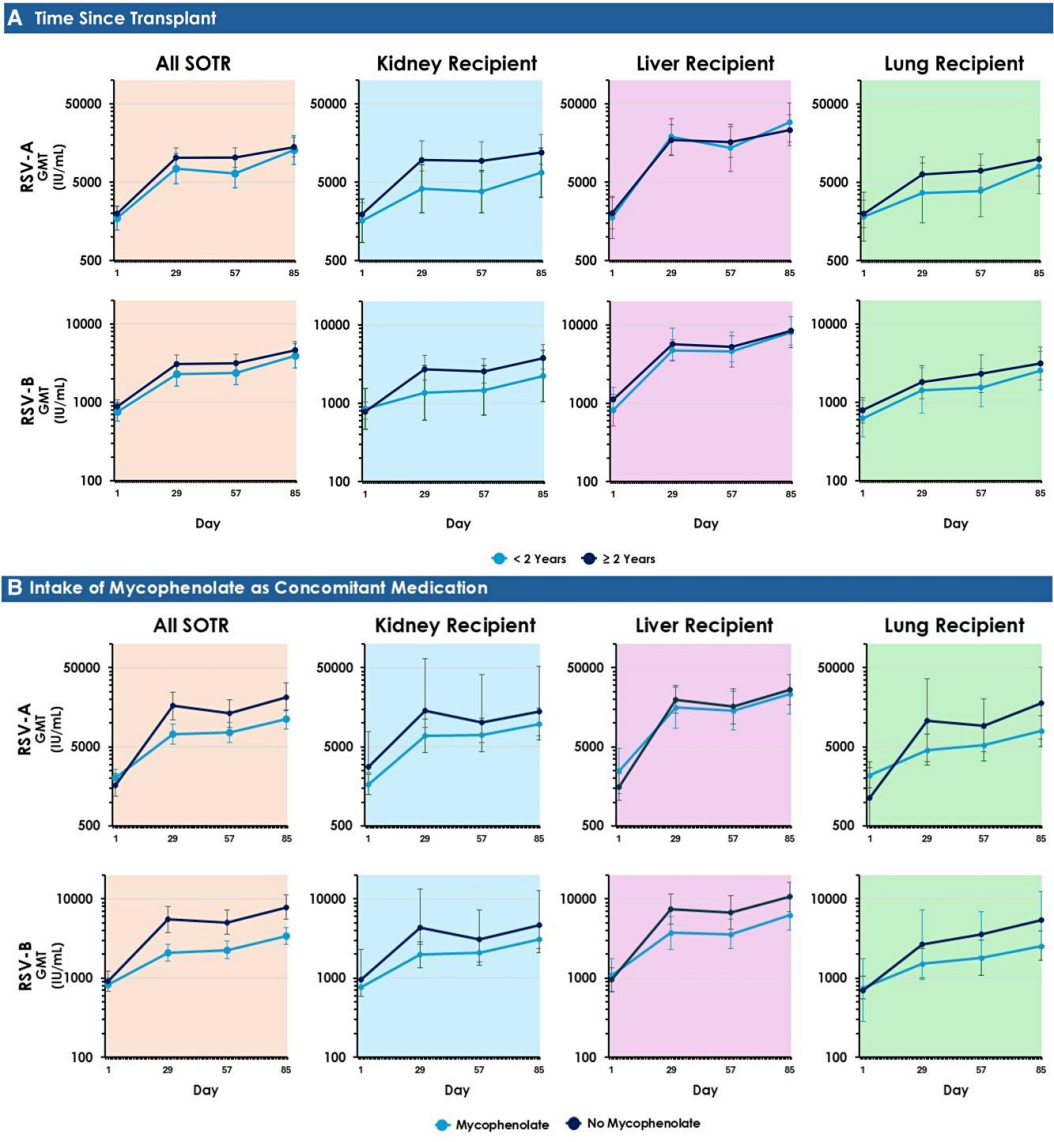
RSV-A and RSV-B neutralizing antibody GMTs by transplant type and time since transplant or mycophenolate use (per-protocol set). GMTs of RSV-A and RSV-B neutralizing antibodies over time in solid organ transplant recipients were stratified by transplant type (all SOTR, kidney, liver, lung; left to right in each panel). The top 2 panels show responses by time since transplant (<2 y vs ≥2 y), and the bottom 2 panels show responses by use of mycophenolate. Antibody values reported as below the LLOQ were replaced by 0.5 × LLOQ. Values above the ULOQ were replaced by the ULOQ. Error bars indicate 95% confidence intervals, calculated using the *t* distribution of log-transformed values and back-transformed for presentation. Stronger relative increases after the second dose (Day 85) were observed among kidney and lung recipients, participants < 2 y post-transplant, and those receiving mycophenolate. Number of participants was as follows: participants by time since transplant: all SOTR < 2 y, n = 39; all SOTR ≥ 2 y, n = 104; kidney recipient < 2 y, n = 11; kidney recipient ≥ 2 y, n = 36; liver recipient < 2 y, n = 16; liver recipient ≥ 2 y, n = 35; lung recipient < 2 y, n = 12; lung recipient ≥ 2 y, n = 33; participants by use of mycophenolate: all SOTRs using mycophenolate, n = 98; all SOTRs not using mycophenolate, n = 45; kidney recipient using mycophenolate, n = 38; kidney recipient not using mycophenolate, n = 9; liver recipient using mycophenolate, n = 24; liver recipient not using mycophenolate, n = 27; lung recipient using mycophenolate, n = 36; lung recipient not using mycophenolate, n = 9. Abbreviations: GMT, geometric mean titer; LLOQ, lower limit of quantification; RSV, respiratory syncytial virus; SOTR, solid organ transplant recipient; ULOQ, upper limit of quantification.

Post hoc analyses were conducted to compare nAb geometric mean ratios between Day 85 and Day 29, which were 1.45 (95% CI, 1.30–1.61) for RSV-A and 1.55 (95% CI, 1.40–1.72) for RSV-B, suggesting a modest increase in response to the second dose. In additional subgroup analyses, greater relative increases after the second dose were observed among kidney and LTRs, participants who were <2 years post-transplant, and those receiving mycophenolate (Figure 4). Participants receiving mycophenolate or <2 years post-transplant had lower GMTs at Day 29 but had more substantial rises by Day 85, reaching levels similar to other subgroups. In contrast, liver transplant recipients had higher GMTs at both Day 29 and Day 85, with more consistent responses across clinical strata, including time since transplant and mycophenolate use. No differences in nAb responses were observed by use of steroids or tacrolimus (data not shown).

Binding antibody responses showed a similar pattern, with marked increases after the first dose and modest further rises after the second, consistent across transplant types and remaining above baseline through Day 181 (Supplementary Tables 6 and 7).

### Cell-Mediated Immunity

**Figure 5.**
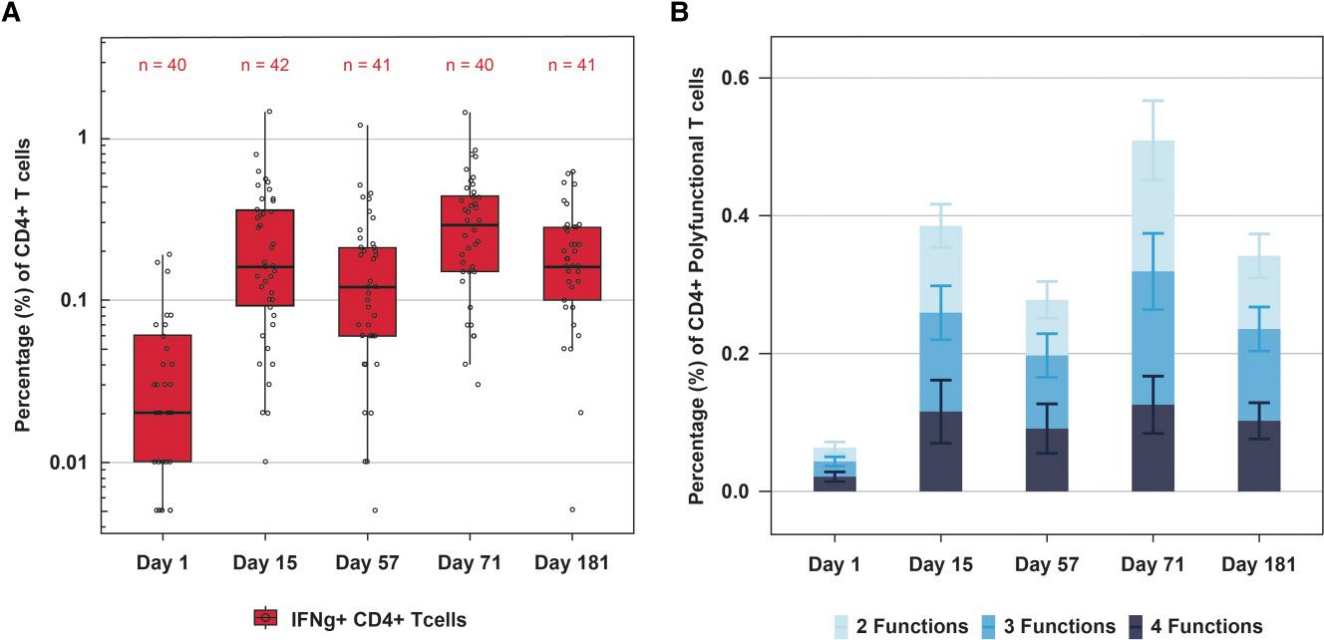
*A*, RSV-F–specific CD4^+^ IFNγ^+^ T-cell responses. *B*, RSV PreF–specific CD4^+^ Th1 polyfunctional responses following mRNA-1345 vaccination in solid organ transplant recipients. RSV-F–specific CD4^+^ T-cell responses expressing IFNγ^+^ (panel *A*) were measured in a subset of solid organ transplant recipients (n = 42; 24 kidney, 8 lung, 8 liver, and 2 combined liver–kidney recipients). Frequency of CD4^+^ non-naive T cells co-expressing 2, 3, or 4 functional markers (IFNγ, IL-2, TNFα, and CD40L) is shown in panel *B*; populations were mutually exclusive and each cell was counted only once according to the highest number of markers expressed. Peripheral blood mononuclear cells were collected and cryopreserved at predefined time points and were stimulated ex vivo with RSV-F peptide pools. Intracellular cytokine staining and flow cytometry were used to quantify functional responses in non-naive CD4^+^ and CD8^+^ T-cell subsets. CD4^+^ IFNγ^+^ responses increased after Dose 1 (Day 15), rose further following Dose 2 (Day 71), and remained elevated through Day 181. A similar trend was observed for CD4^+^ T cells co-expressing 2, 3, or 4 functional markers (panel *B*). Abbreviations: IFN, interferon; IL, interleukin; RSV-F, prefusion F protein of respiratory syncytial virus; Th1, T helper type 1; TNF, tumor necrosis factor.

Cell-mediated immunity was evaluated in 42 participants (24 kidney, 8 lung, 8 liver, and 2 combined liver–kidney recipients). RSV-F–specific CD4⁺ T helper (Th) 1 responses (interferon [IFN]γ⁺, interleukin [IL]-2⁺, tumor necrosis factor [TNF]α⁺) and CD40L⁺ increased after Dose 1 (Day 15), rose further after Dose 2 (Day 71), and remained elevated through Day 181 (Figure 5*A*). Polyfunctional CD4⁺ Th1 responses (co-expressing 2, 3, or all 4 markers) followed a similar pattern (Figure 5*B*). RSV-F–specific CD8⁺ T-cell responses (IFNγ⁺) were detectable by Day 57, increased further by Day 71, and persisted in some participants through Day 181 [19]. CD4⁺ Th2 responses (IL-4⁺, IL-5⁺, IL-13⁺) were minimal, transient, and non-polyfunctional, declining by Day 181 (data not shown). These findings were consistent across transplant types.

## DISCUSSION

In this open-label, phase 3 study, mRNA-1345 (50 µg) was well tolerated and immunogenic in adults ≥ 18 years of age who had received a SOT. The reactogenicity and overall safety profile in this immunocompromised population was consistent with that observed in immunocompetent adults ≥ 60 years from the pivotal phase 3 trial. No vaccine-related discontinuations, deaths, or AEs of special interest were reported.

A single dose of mRNA-1345 was immunogenic across all SOT types, inducing nAb responses against both RSV-A and RSV-B. A second dose administered 56 days later resulted in modest additional increases, and antibody titers remained above baseline through Day 181 across all subgroups. These findings support the potential role of mRNA-1345 in broader immunocompromised populations beyond SOT recipients, including individuals with hematologic malignancies or other causes of immune dysfunction.

Exploratory analyses provided further insight into the immunogenicity of mRNA-1345 in SOT recipients. Post hoc comparisons of GMTs between Days 29 and 85 showed modest increases for both RSV-A and RSV-B. The relative benefit appeared greatest among kidney and lung recipients, those <2 years post-transplant, and participants receiving mycophenolate. In contrast, liver transplant recipients demonstrated higher GMTs at both time points, with more consistent responses across clinical strata. These descriptive findings suggest that, while a single dose of mRNA-1345 is immunogenic across transplant types, some subgroups may benefit from a second dose. Similar findings were reported with the adjuvanted preF vaccine (Arexvy; GlaxoSmithKline), where a second dose boosted responses in transplant recipients to levels observed in healthy older adults approximately 4 months after vaccination, though still lower than peak titers at 1 month post-dose [20]. In contrast, a phase 3 study of the unadjuvanted bivalent preF protein vaccine (Abrysvo; Pfizer) reported no additional increase in antibody titers after Dose 2 given 1 month post vaccination [10]; however, that study included a smaller number of SOT recipients, limiting subgroup analyses. These observations should be interpreted cautiously, as the studies differ in immunocompromised populations, dosing schedules, and vaccine mechanisms.

mRNA-1345 elicited a robust cellular response characterized by Th1-type immunity. RSV-F–specific CD4⁺ T-cell responses, including polyfunctional populations, increased after Dose 1, were further amplified after Dose 2, and remained increased through Day 181. RSV-F–specific CD8⁺ T-cell responses were also detected. Th2 responses were minimal and transient. Given that humoral responses in SOT recipients are often attenuated and heterogeneous, the induction of both CD4⁺ and CD8⁺ T-cell immunity by mRNA-1345 may represent an additional layer of protection, which is particularly important for this vulnerable population.

The reactogenicity and overall safety profile of mRNA-1345 in this trial was consistent with that observed in previous studies, including the pivotal phase 3 trial [15]. Reactogenicity was primarily mild to moderate, and no new vaccine-related safety concerns were identified. Five cases of biopsy-confirmed organ rejection were observed, consistent with the background risk in this population [21, 22]. These cases differed in time of onset, temporal association to mRNA-1345, and underlying pathophysiology, all supporting the investigator assessment that they were not related to vaccination.

nAb responses in liver transplant recipients were comparable to titers associated with protection in immunocompetent adults ≥ 60 years from the pivotal phase 3 trial [14]. While the applicability of this correlate in immunocompromised populations is not fully established, nAbs are recognized as a correlate of protection for RSV disease. This is supported by pediatric data demonstrating that passive immunization, via maternal antibody transfer or monoclonal antibodies such as palivizumab, can prevent RSV illness [23–25]. Additionally, a prespecified correlate of protection analysis based on data from the pivotal efficacy trial for mRNA-1345 demonstrated that Day 29 RSV-A and RSV-B nAb titers met statistical criteria as correlates of protection, with mediation analysis indicating that most of the observed vaccine efficacy was attributable to the nAb response [18].Thus, it is reassuring to observe robust nAb responses in this severely immunocompromised population. In addition, the induction of cell-mediated immunity may further contribute to protection, especially in participants who mount lower or more variable humoral responses.

Strengths of the study are the inclusion of participants across 3 SOT types, with robust representation of LTRs, a population with profound immunosuppression that is often underrepresented in vaccine trials. Additionally, eligibility criteria allowed vaccination as early as 180 days post-transplant, and approximately one-quarter of participants were within 2 years of transplantation, reflecting clinical scenarios in which RSV vaccination may be considered. The study also assessed the durability of immune responses, including both nAbs and RSV-F–specific T-cell responses, through Day 181.

Limitations of this study include the absence of a control group or single-dose comparator, which prevents definitive conclusions about the incremental benefit of the second dose. All immunogenicity endpoints were descriptive, and no formal efficacy evaluation was performed. Although the overall sample size was consistent with other studies of SOT recipients and allowed for subgroup analyses across transplant types, ongoing follow-up will be important to assess durability of responses and longer-term safety of 1 and 2 doses of mRNA-1345.

In summary, mRNA-1345 was well tolerated and immunogenic in adult SOT recipients. A single dose elicited nAb responses across all subgroups, with potential additional benefit from a second dose in certain participants. The durable nAb responses and induction of robust CD4⁺ polyfunctional cellular responses provide further immunologic support for the potential benefit of RSV vaccination in this population. These findings suggest that mRNA-1345 may be a valuable preventive tool against RSV in immunocompromised adults.

## Supplementary Material

ciag108_Supplementary_Data

## Supplementary Data

Supplementary materials are available at *Clinical Infectious Diseases* online. Consisting of data provided by the authors to benefit the reader, the posted materials are not copyedited and are the sole responsibility of the authors, so questions or comments should be addressed to the corresponding author.

## Notes

***Author contributions.*** Authors contributed toward the concept and design of the study, were involved in data collection, and/or analyzed or interpreted the data. All authors provided writing, review, or intellectual contributions to the work and approved the final draft.

***Acknowledgments.*** The authors thank the participants in the trial and their families, site personnel, and all members of the study. They acknowledge the contributions of Avi Collins, BScN; Rituparna Das, MD, PhD; Christina Grassi, MD; Jaya Goswami, MD; Xiaolin Chang, PhD; Hsiaohsuan Kuo, PhD; Jenni Mou, PhD; Caroline Reuter, MD; Anthony Rizk, PharmD; Sonia Stoszek, PhD; Jennifer Tu, PhD; Eleanor Wilson, MD; and Honghong Zhou, PhD. CMI Data were generated by the Clinical Cytometry Group, Moderna Clinical Biomarker Laboratories (CBL), led by Paul C. Trampont, with support from CBL-Operations, led by Hassana Fathallah, and by CBL Compliance, led by Jennifer Biroschak. The authors further recognize that many additional colleagues contributed to the successful execution of this study, and they extend their appreciation to all individuals whose efforts supported this work. Medical writing and editorial assistance were provided by Alana Simorellis, PhD, and Barbara Kuter, PhD, MPH, of Moderna, Inc., and Agnieszka Looney, PhD, of MEDiSTRAVA, in accordance with Good Publication Practice (GPP3) guidelines, funded by Moderna, Inc., and under the direction of authors.

***Data sharing.*** Access to participant-level data presented in this article and supporting clinical documents with external researchers who provide methodologically sound scientific proposals will be available upon reasonable request for products or indications that have been approved by regulators in the relevant markets and subject to review from 24 months after study completion. Such requests can be made to Moderna, Inc., 325 Binney St, Cambridge, Massachusetts 02142 (data_sharing@modernatx.com). A material transfer and/or data access agreement with the sponsor will be required for accessing shared data. All other relevant data are presented in the paper.

***Trademark statement.*** mRESVIA is a trademark of Moderna.

***Financial support.*** This work was supported by Moderna, Inc.

## References

[1] Falsey AR, Hennessey PA, Formica MA, Cox C, Walsh EE. Respiratory syncytial virus infection in elderly and high-risk adults. N Engl J Med 2005; 352:1749–59. 15858184 10.1056/NEJMoa043951

[2] Falsey AR, Walsh EE. Respiratory syncytial virus infection in adults. Clin Microbiol Rev 2000; 13:371–84. 10885982 10.1128/cmr.13.3.371-384.2000 PMC88938

[3] Prasad N, Walker TA, Waite B, et al Respiratory syncytial virus–associated hospitalizations among adults with chronic medical conditions. Clin Infect Dis 2021; 73:e158–63. 32531019 10.1093/cid/ciaa730

[4] Villanueva DH, Arcega V, Rao M. Review of respiratory syncytial virus infection among older adults and transplant recipients. Ther Adv Infect Dis 2022; 9:20499361221091413. 35464624 10.1177/20499361221091413 PMC9019318

[5] Abbas S, Raybould JE, Sastry S, de la Cruz O. Respiratory viruses in transplant recipients: more than just a cold. Clinical syndromes and infection prevention principles. Int J Infect Dis 2017; 62:86–93. 28739424 10.1016/j.ijid.2017.07.011

[6] Liu C, Ho DY, Boeckh M. Respiratory viral infections in transplant recipients. New York: Springer, 2018.

[7] Solidoro P, Curtoni A, Minuto S, et al Impact of RSV infection in transplant and immunocompromised population: incidence and co-infections: retrospective analysis of a single centre. J Clin Med 2025; 14:4803. 40649177 10.3390/jcm14134803 PMC12251039

[8] Weigt SS, Gregson AL, Deng JC, Lynch JP 3rd, Belperio JA. Respiratory viral infections in hematopoietic stem cell and solid organ transplant recipients. Semin Respir Crit Care Med 2011; 32:471–93. 21858751 10.1055/s-0031-1283286 PMC4209842

[9] Fry SE, Terebuh P, Kaelber DC, Xu R, Davis PB. Effectiveness and safety of respiratory syncytial virus vaccine for US adults aged 60 years or older. JAMA Netw Open 2025; 8:e258322. 40343698 10.1001/jamanetworkopen.2025.8322 PMC12065041

[10] Almeida NC, Parameswaran L, DeHaan EN, et al Immunogenicity and safety of the bivalent respiratory syncytial virus prefusion F subunit vaccine in immunocompromised or renally impaired adults. Vaccines (Basel) 2025; 13:328. 40266222 10.3390/vaccines13030328 PMC11946143

[11] Moderna . Moderna receives U.S. FDA approval for RSV vaccine mRESVIA(R). Available at: https://investors.modernatx.com/news/news-details/2024/Moderna-Receives-U.S.-FDA-Approval-for-RSV-Vaccine-mRESVIAR/default.aspx. Accessed 5 June 2024.

[12] Moderna. mRESVIA® (Respiratory Syncytial Virus Vaccine). Prescribing information. Available at: https://www.modernatx.com/en-US/products/mresvia. Accessed 12 September 2024.

[13] Moderna . Moderna receives European Commission approval for RSV vaccine mRESVIA(R). Available at: https://s29.q4cdn.com/435878511/files/doc_news/Moderna-Receives-European-Commission-Approval-for-RSV-Vaccine-mRESVIAR-2024.pdf. Accessed 28 January 2025.

[14] Goswami J, Baqui AH, Doreski PA, et al Humoral immunogenicity of mRNA-1345 RSV vaccine in older adults. J Infect Dis 2024; 230:e996–1006. 38889247 10.1093/infdis/jiae316 PMC11566230

[15] Wilson E, Goswami J, Baqui AH, et al Efficacy and safety of an mRNA-based RSV preF vaccine in older adults. N Engl J Med 2023; 389:2233–44. 38091530 10.1056/NEJMoa2307079

[16] Mayer EF, Falsey AR, Clark R, et al Safety, tolerability, and immunogenicity of mRNA-1345 in adults at increased risk for RSV disease aged 18 to 59 years. Clin Infect Dis 2025; 81:e708–16. 10.1093/cid/ciaf292 40464662

[17] Figueroa AL, Azzi JR, Eghtesad B, et al Safety and immunogenicity of the mRNA-1273 coronavirus disease 2019 vaccine in solid organ transplant recipients. J Infect Dis 2024; 230:e591–600. 38513368 10.1093/infdis/jiae140 PMC11420796

[18] Ma C, Du J, Lan L, et al Immune correlates analysis of mRNA-1345 RSV vaccine efficacy clinical trial. Nat Commun 2025; 16:6118. 40610413 10.1038/s41467-025-61153-x PMC12229610

[19] Mayer EF, Wolfe CR, Falsey A, et al T-cell responses following one and two doses of mRNA-1345 RSV vaccine in adult solid organ transplant recipients. Presented at: IDWeek. Atlanta, GA, October 19-22, 2025.

[20] Gerber S. Immune response and safety among adult lung and kidney transplant recipients. Available at: https://www.cdc.gov/acip/downloads/slides-2024-10-23-24/04-RSV-Adult-Gerber-508.pdf. Accessed 18 September 2025.

[21] Testaert H, Bouet M, Valour F, et al Incidence, management and outcome of respiratory syncytial virus infection in adult lung transplant recipients: a 9-year retrospective multicentre study. Clin Microbiol Infect 2021; 27:897–903. 32827713 10.1016/j.cmi.2020.07.050

[22] Finlen Copeland CA, Snyder LD, Zaas DW, Turbyfill WJ, Davis WA, Palmer SM. Survival after bronchiolitis obliterans syndrome among bilateral lung transplant recipients. Am J Respir Crit Care Med 2010; 182:784–9. 20508211 10.1164/rccm.201002-0211OC PMC2949403

[23] Palivizumab, a humanized respiratory syncytial virus monoclonal antibody, reduces hospitalization from respiratory syncytial virus infection in high-risk infants. The IMpact-RSV Study Group. Pediatrics 1998; 102:531–7. 9724660

[24] O'Brien KL, Chandran A, Weatherholtz R, et al Efficacy of motavizumab for the prevention of respiratory syncytial virus disease in healthy Native American infants: a phase 3 randomised double-blind placebo-controlled trial. Lancet Infect Dis 2015; 15:1398–408. 26511956 10.1016/S1473-3099(15)00247-9

[25] Buchwald AG, Graham BS, Traore A, et al Respiratory syncytial virus (RSV) neutralizing antibodies at birth predict protection from RSV illness in infants in the first 3 months of life. Clin Infect Dis 2021; 73:e4421-e7. 32463443 10.1093/cid/ciaa648 PMC8662775

