# Supplementary material for: Respiratory Syncytial Virus Vaccination in Solid Organ Transplant Recipients: Interim Findings From a Phase 3 Trial of mRNA-1345
Source: Clin Infect Dis. 2026 Feb 18;82(5):e1112–21. doi: 10.1093/cid/ciag108 (PMC13189680; doi:10.1093/cid/ciag108)
Supplement: ciag108_Supplementary_Data [file ciag108_supplementary_data.docx]

# Supplementary Appendix

Supplement to: RSV Vaccination in Solid Organ Transplant Recipients: Interim Findings From a Phase 3 Trial of mRNA-1345

This appendix has been provided by the authors to give readers additional information about the work.

## List of Trial Investigators

| **Investigator** | **Site Name** | **Location** |
| --- | --- | --- |
| Kabbani, Dima | University of Alberta | Alberta, Alberta, Canada |
| McNeil, Shelly | Canadian Center for Vaccinology | Halifax, Nova Scotia, Canada |
| Clark, Rebecca | Layton Medical Centre | Blackpool, Lancashire, United Kingdom |
| Soiza, Roy | Aberdeen Royal Infirmary - PPDS | Aberdeen, Scotland, United Kingdom |
| Sheridan, Raymond (Ray) | Royal Devon and Exeter Hospital (Wonford) - Barrack Rd | Exeter, Devon, United Kingdom |
| Burns, Fiona | The Royal Free Hospital | London, City of London, United Kingdom |
| Falsey, Ann | University of Rochester - Rochester General Hospital - PPDS | Rochester, New York, USA |
| Nathan, Richard | Snake River Research, PLLC | Idaho Falls, Idaho, USA |
| Cardona, Jose | Indago Research and Health Center | Hialeah, Florida, USA |
| Myneni, Banu | Velocity Clinical Research - Family Practice - Portsmouth - PPDS | Suffolk, Virginia, USA |
| Schaenman, Joanna | David Geffen School of Medicine at UCLA | Los Angeles, California, USA |
| Rivard, Rebecca | DM Clinical Research - Southfield - ERN - PPDS | Southfield, Michigan, USA |
| Balboul, Rana | DM Clinical Research - Southfield - ERN - PPDS | Southfield, Michigan, USA |
| Patel, Nathan | DM Clinical Research - Southfield - ERN - PPDS | Southfield, Michigan, USA |
| Wheeler, David | DM Clinical Research - Philadelphia - ERN - PPDS | Philadelphia, Pennsylvania, USA |
| Yenal, Kem | DM Clinical Research - Philadelphia - ERN - PPDS | Philadelphia, Pennsylvania, USA |
| Ogbuagu, Onyema | Yale University School of Medicine - 135 College St | New Haven, Connecticut, USA |
| McCort, Margaret | Montefiore Medical Center - BRANY - PPDS | Bronx, New York, USA |
| Irfan, Muhammad | Cyfair Clinical Research Center - ERN - PPDS | Houston, Texas, USA |
| Czapka, Michael | University of Chicago | Chicago, Illinois, USA |
| Wolfe, Cameron | Duke University Medical Center - 2301 Erwin Dr | Durham, North Carolina, USA |
| Piscitelli, Thomas | Velocity Clinical Research - Hampton - PPDS | Hampton, Virginia, USA |
| McCormick, Brian | Velocity Clinical Research - Hampton - PPDS | Hampton, Virginia, USA |
| Titone, Dominic | Critical Care, Pulmonary and Sleep Associates / CCT Research | Lakewood, Colorado, USA |
| Herc, Erick | Henry Ford Hospital | Detroit, Michigan, USA |
| Ramesh, Mayer | Henry Ford Hospital | Detroit, Michigan, USA |
| Wijewardane, Priyantha N. | Baptist Health Center for Clinical Research | Little Rock, Arkansas, USA |

## Supplementary Methods

## Trial Design

Part B of Study P303 is a phase 3, open-label, single-arm, multicenter study. The primary purpose of Study P303 Part B was to establish the safety, tolerability, and immunogenicity of mRNA-1345 injection in immunocompromised adults who received solid organ transplant (SOT). The study employed a single-arm design to facilitate operational execution and ensure sufficient participant numbers across key SOT subgroups. Immunogenicity assessments were conducted after each dose and longitudinally to characterize both the magnitude and durability of immune responses in this high-risk population. This study was conducted in multiple centers in the United States, Canada, and the United Kingdom. Moderna was responsible for the overall trial design, site selection, monitoring, and data analysis, which was facilitated by PPD, Inc. (Wilmington, NC, USA). All authors agreed to submit the manuscript for publication. Medical writers who were funded by the sponsor assisted with the development of the manuscript.

## Study Inclusion Criteria

Participants were eligible to be included in the trial only if all the following criteria applied:

**Age**

1. Adults ≥18 years of age at the time of consent who are primarily responsible for self-care

and activities of daily living.

**Type of Participant and Disease Characteristics**

2. Recipient of a SOT (kidney, liver, or lung transplant) ≥180 days prior to the day of consent and receiving chronic immunosuppressive therapy for the prevention of allograft rejection.

3. Participant was willing and able (on both a physical and cognitive basis) to give written informed consent prior to trial enrollment.

4. Was able to comply with trial requirements, including access to transportation for trial visits and telephone communication with trial staff.

5. Female participants of non-childbearing potential could be enrolled in the trial. Non-childbearing potential was defined as postmenopausal or permanently sterilized. The follicle-stimulating hormone level could have been measured at the discretion of the Investigator to confirm postmenopausal status.

6. Female participants of childbearing potential could be enrolled in the trial if the participant fulfilled all the following criteria:

a. Had a negative pregnancy test at the screening visit and on the day of first injection (Day 1).

b. Had practiced adequate contraception or had abstained from all activities that could result in pregnancy for at least 28 days prior to Day 1.

c. Had agreed to continue adequate contraception through 90 days following the second injection. Adequate female contraception was defined as consistent and correct use of a local health authority–approved contraceptive method in accordance with the product label.

d. Was not currently breastfeeding.

## Study Exclusion Criteria

Participants were excluded from the trial if any of the following criteria applied:

1. Previous treatment with:

a. Alemtuzumab or rituximab within 2 years prior to Day 1.

b. Plasmapheresis within 30 days prior to Day 1.

2. A history of complications of immunosuppression; specifically, the following:

a. Replacement immunoglobulin within 30 days prior to Day 1.

b. Under investigation or active treatment for PTLD or another malignancy.

c. Active treatment for CMV viremia within 30 days prior to Day 1.

d. Active treatment for BK viremia within 30 days prior to Day 1.

e. Other symptomatic viral infections.

3. A history of biopsy-proven or clinically diagnosed rejection within 90 days prior to Day 1 or suspected active chronic rejection according to the Investigator’s judgment.

4. Participation in another clinical research trial where the participant had received an investigational product (drug/biologic/device) within 180 days prior to Day 1. Participation in an RSV trial at any time prior to Day 1 was exclusionary.

5. History of a diagnosis or condition, within 30 days prior to Day 1, that, in the judgment of the Investigator, was clinically unstable or could have affected participant safety, assessment of safety endpoints, assessment of immune response, or adherence to trial procedures. Clinically unstable was defined as follows:

a. Diagnosis or condition requiring significant changes in management or medication and included ongoing workup of an undiagnosed illness that could have led to a new diagnosis or condition; and

b. Changes in medical therapy due to treatment failure or toxicity; and

c. Significant worsening, in the opinion of the Investigator, of a medical condition.

Note: HIV-positive participants with CD4 count ≥350 cells/mm³ and an undetectable HIV viral load within the past year (low-level variations from 50-500 viral copies that did not lead to changes in antiretroviral therapy), as determined from the participant’s medical records, were permitted.

6. Dermatologic conditions that could have affected local solicited AR assessments (e.g., tattoos, psoriasis patches affecting the skin over the deltoid areas).

7. Reported history of anaphylaxis or severe hypersensitivity reaction after receipt of the mRNA-1345 injection or any components of the mRNA-1345 injection.

8. Reported history of a bleeding disorder that was considered a contraindication to intramuscular injection or phlebotomy.

9. History of a serious reaction to any prior vaccination.

10. History of Guillain-Barré syndrome.

11. Had received or planned to receive any non-trial vaccine (including authorized or approved vaccines for the prevention of COVID-19) within 28 days before or after trial injections (on Day 1 and Day 57). Non-trial vaccine(s) were not delayed.

12. Had received or planned to receive any authorized or approved vaccine for the prevention of RSV.

13. Was acutely ill or febrile (temperature ≥38.0 °C/≥100.4 °F) within 72 hours prior to or at the screening visit or Day 1. Participants meeting this criterion could have been rescheduled within the 5-day screening window and retained their initially assigned participant number.

14. Any medical, psychiatric, or occupational condition, including a reported history of drug or alcohol abuse, that, in the opinion of the Investigator, might have posed additional risk due to participation in the trial or could have interfered with the interpretation of trial results.

15. Known uncontrolled disorder of coagulation. Note: Participants receiving aspirin, clopidogrel, prasugrel, dipyridamole, dabigatran, apixaban, rivaroxaban, or warfarin for cardiovascular prophylaxis or prophylaxis of thromboembolic disease or stroke in the setting of atrial fibrillation and under good control were not excluded.

16. History of myocarditis, pericarditis, or myopericarditis.

17.Donated ≥450 mL of blood products <28 days prior to Day 1.

18. Was a member of trial personnel or an immediate family member or household member of trial personnel.

## Statistical Analysis

Analysis was descriptive and there was no hypothesis testing in Part B of Study P303.

The sample size was not based on any formal hypotheses. With 150 participants receiving study injection, there was a probability of >95% to observe at least 1 participant with an AE, if the true incidence rate of the AE was 3%; if the true incidence rate was 1%, the probability of observing at least 1 participant with an AE was approximately 77.9%.

Analysis was descriptive and there was no hypothesis testing in Part B of Study P303.

Determination of Sample Size

The sample size was not based on any formal hypotheses. With 150 participants receiving the study injection, there was a probability of >95% to observe at least 1 participant with an AE, if the true incidence rate of the AE was 3%; if the true incidence rate was 1%, the probability of observing at least 1 participant with an AE was approximately 77.9%.

Analysis sets used for statistical analyses are presented below:

Study P303 Part B Analysis Sets

| **Analysis Set** | **Description** |
| --- | --- |
| Enrolled set | The enrolled set consisted of all participants who were enrolled in the study, regardless of the participant’s treatment status in the study. Participants were included in the study intervention group to which they were assigned. |
| Full analysis set (FAS) | All participants who received any study injection. Participants were included in the study intervention group to which they are assigned. |
| Per-protocol (PP) set | Included all participants in the FAS who received the assigned injection dose according to the protocol, complied with immunogenicity blood sampling to have a baseline and at least 1 post-injection assessment, and had no significant protocol deviations that impacted the immune response. Participants who used the medication listed in the exclusion criteria were excluded. The PP set was the primary population used for immunogenicity data. Participants were included in the study intervention group to which they were assigned. |
| Solicited safety set | Included all participants who received any study injection and contributed any solicited AR data. The solicited safety set was used for the analyses of solicited ARs, and participants were included in the study intervention group corresponding to the injection they actually received. |
| Safety set | Included all participants who received any study injection. Participants were included in the study intervention group corresponding to the injection they actually received for the analysis of safety data using the safety set. |

Abbreviations: AR, adverse reaction; FAS, full analysis set; PP, per-protocol.

Immunogenicity Analyses

Immunogenicity analyses were based on the Study P303 Part B PP set.

Serum neutralizing antibodies to RSV-A and RSV-B were measured using validated microneutralization assays. Neutralizing antibody titers were converted to international units per milliliter (IU/mL) using conversion factors derived from the World Health Organization (WHO) international standard antiserum for RSV-A and RSV-B (2020).

Serum binding antibodies against the RSV prefusion F (preF) protein were measured using a validated quantitative multiplex assay based on Luminex® technology, with results expressed as geometric mean concentrations (GMCs) in arbitrary units per milliliter (AU/mL).

The primary immunogenicity endpoints, geometric mean titers (GMTs) of RSV-A and RSV-B neutralizing antibodies (nAbs) on Day 85 with corresponding 95% confidence intervals (CIs) were provided. The 95% CI of GMT was calculated based on the *t* distribution of the log-transformed values and then back-transformed to the to the original scale.

The number and percentage of participants with seroresponse at each timepoint were provided with 2-sided 95% CIs using the Clopper-Pearson method.

In addition, the GMT of RSV-A and RSV-B nAbs and geometric mean concentration of prefusion (preF) binding antibodies (bAbs) with the corresponding 95% CIs were provided at each timepoint. The 95% CIs of GMTs were calculated based on the *t* distribution of the log-transformed values and then back-transformed to the original scale. The GMFRs of RSV-A, RSV-B nAbs, and preF bAbs with the corresponding 95% CIs at each post-baseline timepoint over pre-injection baseline were provided. Descriptive statistics (including 95% CIs) of the immunogenicity endpoints were also provided.

For summarizations of GMTs/geometric mean concentrations, antibody titers/concentrations reported as below the lower limit of quantification (LLOQ) were replaced by 0.5 × LLOQ. Values that were greater than the upper limit of quantification (ULOQ) were converted to the ULOQ, unless otherwise specified.

The seroresponse rates (SRRs) of RSV-A nAbs, RSV-B nAbs, and RSV preF bAbs were defined as the proportion of participants with post-injection titers ≥4 × LLOQ if baseline was <LLOQ or a ≥4-fold increase from baseline if baseline was ≥LLOQ.

The proportion of participants with seroresponse and ≥2-fold in RSV-A nAb titers, RSV-B nAb titers, and RSV preF bAbs from baseline were provided with 2-sided 95% CIs using the Clopper-Pearson method by post-baseline timepoint.

Descriptive statistics of the immunogenicity endpoints were provided by the following the subgroups of SOT type (lung, liver, kidney).

If the numbers of participants in certain subgroups were too small, they may have been combined with other subgroups for the subgroup analyses.

***Ex vivo* Stimulation and Intracellular Staining Assay**

Peripheral blood mononuclear cell (PBMC) samples were prepared from heparin whole blood specimens using standard ficoll separation methods and were cryopreserved within 8 hours of blood draw. Samples were stored in liquid nitrogen vapor phase until testing. Cryopreserved PBMCs were thawed and rested overnight in a CO2 incubator. PBMCs were ex vivo stimulated by incubating with DMSO or RSV Pre-F peptide pool (GeneScript Biotech Corp; Piscataway, NJ, USA; 15mers overlapping by 11 amino acids) and fluorescently labeled CD107a for 6 hours with protein transport inhibitor. PBMC samples were stained with fluorescently labeled antibodies for cell surface (CD4, CD8 CD14, CD16, CD19, CD56, CD45RA, CCR7, and TCRγδ) and intracellular (CD3, CD69, CD154, IL-2, IL-4, IL-5, IL-13, IL17a, IFN-γ, TNF-α, and Granzyme B) markers of functional T-cell responses and acquired on a 5-laser Aurora spectral flow cytometer (Cytek*®* Biosciences; Fremont, CA, USA). Data were analyzed using FCS-Express™ 7 IVD (De Novo Software; Pasadena, CA, USA). DMSO functional responses were subtracted from RSV responses. Only DMSO corrected responses are shown.

# Supplementary Tables

## Supplementary Table 1. Summary of Solicited Adverse Reactions by Toxicity Grade (Solicited Safety Set)

|  | **mRNA-1345 50 µg** | |
| --- | --- | --- |
| **Solicited Adverse Reaction   Category   Grade** | **Within 7 Days After  First Injection**  **(N = 150) n (%)** | **Within 7 Days After  Second Injection (N = 146) n (%)** |
|  | |  |
| Solicited adverse reactions - N1 | 150 | 146 |
| Any solicited adverse reactions | 125 (83.3) | 122 (83.6) |
| 95% CI | 76.4, 88.9 | 76.5, 89.2 |
| Grade 1 | 73 (48.7) | 55 (37.7) |
| Grade 2 | 46 (30.7) | 55 (37.7) |
| Grade 3 | 6 (4.0) | 11 (7.5) |
| Grade 4 | 0 | 1 (0.7) |
| Grade 3 or grade 4 | 6 (4.0) | 12 (8.2) |
|  | |  |
| Solicited local adverse reactions - N1 | 150 | 146 |
| Any solicited local adverse reactions | 111 (74.0) | 113 (77.4) |
| 95% CI | 66.2, 80.8 | 69.7, 83.9 |
| Grade 1 | 77 (51.3) | 73 (50.0) |
| Grade 2 | 33 (22.0) | 38 (26.0) |
| Grade 3 | 1 (0.7) | 2 (1.4) |
| Grade 4 | 0 | 0 |
| Grade 3 or grade 4 | 1 (0.7) | 2 (1.4) |
|  | |  |
| Pain - N1 | 150 | 146 |
| Any | 111 (74.0) | 112 (76.7) |
| Grade 1 | 79 (52.7) | 73 (50.0) |
| Grade 2 | 31 (20.7) | 37 (25.3) |
| Grade 3 | 1 (0.7) | 2 (1.4) |
| Grade 4 | 0 | 0 |
| Grade 3 or grade 4 | 1 (0.7) | 2 (1.4) |
|  | |  |
| Erythema (redness) - N1 | 150 | 146 |
| Any | 3 (2.0) | 1 (0.7) |
| Grade 1 | 1 (0.7) | 1 (0.7) |
| Grade 2 | 2 (1.3) | 0 |
| Grade 3 | 0 | 0 |
| Grade 4 | 0 | 0 |
| Grade 3 or grade 4 | 0 | 0 |
|  | |  |
| Swelling (hardness) - N1 | 150 | 146 |
| Any | 4 (2.7) | 1 (0.7) |
| Grade 1 | 4 (2.7) | 1 (0.7) |
| Grade 2 | 0 | 0 |
| Grade 3 | 0 | 0 |
| Grade 4 | 0 | 0 |
| Grade 3 or grade 4 | 0 | 0 |
|  | |  |
| Axillary (underarm) swelling or tenderness - N1 | 150 | 146 |
| Any | 19 (12.7) | 25 (17.1) |
| Grade 1 | 15 (10.0) | 19 (13.0) |
| Grade 2 | 4 (2.7) | 6 (4.1) |
| Grade 3 | 0 | 0 |
| Grade 4 | 0 | 0 |
| Grade 3 or grade 4 | 0 | 0 |
|  | |  |
| Solicited systemic adverse reactions - N1 | 150 | 146 |
| Any solicited systemic adverse reactions | 96 (64.0) | 94 (64.4) |
| 95% CI | 55.8, 71.7 | 56.0, 72.1 |
| Grade 1 | 60 (40.0) | 41 (28.1) |
| Grade 2 | 30 (20.0) | 42 (28.8) |
| Grade 3 | 6 (4.0) | 10 (6.8) |
| Grade 4 | 0 | 1 (0.7) |
| Grade 3 or grade 4 | 6 (4.0) | 11 (7.5) |
|  | |  |
| Fever - N1 | 150 | 146 |
| Any | 4 (2.7) | 6 (4.1) |
| Grade 1 | 1 (0.7) | 3 (2.1) |
| Grade 2 | 3 (2.0) | 1 (0.7) |
| Grade 3 | 0 | 2 (1.4) |
| Grade 4 | 0 | 0 |
| Grade 3 or grade 4 | 0 | 2 (1.4) |
|  | |  |
| Headache - N1 | 150 | 146 |
| Any | 56 (37.3) | 59 (40.4) |
| Grade 1 | 43 (28.7) | 38 (26.0) |
| Grade 2 | 12 (8.0) | 18 (12.3) |
| Grade 3 | 1 (0.7) | 3 (2.1) |
| Grade 4 | 0 | 0 |
| Grade 3 or grade 4 | 1 (0.7) | 3 (2.1) |
|  | |  |
| Fatigue - N1 | 150 | 146 |
| Any | 66 (44.0) | 68 (46.6) |
| Grade 1 | 39 (26.0) | 32 (21.9) |
| Grade 2 | 23 (15.3) | 30 (20.5) |
| Grade 3 | 4 (2.7) | 6 (4.1) |
| Grade 4 | 0 | 0 |
| Grade 3 or grade 4 | 4 (2.7) | 6 (4.1) |
|  | |  |
| Myalgia - N1 | 150 | 146 |
| Any | 45 (30.0) | 51 (34.9) |
| Grade 1 | 35 (23.3) | 28 (19.2) |
| Grade 2 | 8 (5.3) | 21 (14.4) |
| Grade 3 | 2 (1.3) | 2 (1.4) |
| Grade 4 | 0 | 0 |
| Grade 3 or grade 4 | 2 (1.3) | 2 (1.4) |
|  | |  |
| Arthralgia - N1 | 150 | 146 |
| Any | 31 (20.7) | 43 (29.5) |
| Grade 1 | 24 (16.0) | 27 (18.5) |
| Grade 2 | 6 (4.0) | 14 (9.6) |
| Grade 3 | 1 (0.7) | 1 (0.7) |
| Grade 4 | 0 | 1 (0.7) |
| Grade 3 or grade 4 | 1 (0.7) | 2 (1.4) |
|  | |  |
| Nausea/Vomiting - N1 | 150 | 146 |
| Any | 18 (12.0) | 23 (15.8) |
| Grade 1 | 12 (8.0) | 19 (13.0) |
| Grade 2 | 6 (4.0) | 3 (2.1) |
| Grade 3 | 0 | 1 (0.7) |
| Grade 4 | 0 | 0 |
| Grade 3 or grade 4 | 0 | 1 (0.7) |
|  | |  |
| Chills - N1 | 150 | 146 |
| Any | 20 (13.3) | 31 (21.2) |
| Grade 1 | 14 (9.3) | 20 (13.7) |
| Grade 2 | 6 (4.0) | 11 (7.5) |
| Grade 3 | 0 | 0 |
| Grade 4 | 0 | 0 |
| Grade 3 or grade 4 | 0 | 0 |

Abbreviations: any, grade 1 or above; CI, confidence interval.

N1 is the number of exposed participants who submitted any data for the event.

Percentages were based on the number of exposed participants who submitted any data for the event (N1).

Within each category of summary, a participant was counted once with the highest toxicity grade within 7 days.

95% confidence interval was calculated using the Clopper-Pearson method.

## Supplementary Table 2. Summary of Characteristics of Solicited Adverse Reactions Within 7 Days After First and Second Injections (Solicited Safety Set)

|  | | **mRNA-1345 50 µg** | |
| --- | --- | --- | --- |
|  | **Within 7 Days After  First Injection**  **(N = 150) n (%)** | | **Within 7 Days After  Second Injection (N = 146) n (%)** |
|  | | |  |
| Solicited adverse reactions - N1 | | 150 | 146 |
| Any solicited adverse reactions | | 125 (83.3) | 122 (83.6) |
| Day of onset, median (min, max) | | 1.0 (1, 6) | 1.0 (1, 7) |
| Duration (days), median (min, max) | | 3.0 (1, 20) | 3.0 (1, 102) |
| Solicited local adverse reactions - N1 | | 150 | 146 |
| Any solicited adverse reactions | | 111 (74.0) | 113 (77.4) |
| Day of onset, median (min, max) | | 2.0 (1, 3) | 2.0 (1, 4) |
| Duration (days), median (min, max) | | 2.0 (1, 9) | 2.0 (1, 7) |
| Pain | |  |  |
| Any solicited adverse reactions | | 111 (74.0) | 112 (76.7) |
| Day of onset, median (min, max) | | 2.0 (1, 3) | 2.0 (1, 4) |
| Duration, (days), median (min, max) | | 2.0 (1, 9) | 2.0 (1, 7) |
| Erythema | |  |  |
| Any solicited adverse reactions | | 3 (2.0) | 1 (0.7) |
| Day of onset, median (min, max) | | 3.0 (2, 3) | 1.0 (1, 1) |
| Duration, (days), median (min, max) | | 3.0 (2, 5) | 1.0 (1, 1) |
| Swelling | |  |  |
| Any solicited adverse reactions | | 4 (2.7) | 1 (0.7) |
| Day of onset, median (min, max) | | 3.5 (3, 6) | 2.0 (2, 2) |
| Duration (days), median (min, max) | | 1.5 (1, 2) | 1.0 (1, 1) |
| Axillary Swelling | |  |  |
| Any solicited adverse reactions | | 19 (12.7) | 25 (17.1) |
| Day of onset, median (min, max) | | 2.0 (1, 7) | 2.0 (1, 7) |
| Duration (days), median (min, max) | | 1.0 (1, 6) | 1.0 (1, 6) |
| Solicited systemic adverse reactions - N1 | | 150 | 146 |
| Any solicited adverse reactions | | 96 (64.0) | 94 (64.4) |
| Day of onset, median (min, max) | | 2.0 (1, 6) | 2.0 (1, 7) |
| Duration (days), median (min, max) | | 2.5 (1, 20) | 3.0 (1, 102) |
| Fever | |  |  |
| Any solicited adverse reactions | | 4 (2.7) | 6 (4.1) |
| Day of onset, median (min, max) | | 4.0 (2, 7) | 2.5 (2, 4) |
| Duration (days), median (min, max) | | 1.0 (1, 1) | 1.0 (1, 2) |
| Headache | |  |  |
| Any solicited adverse reactions | | 56 (37.3) | 59 (40.4) |
| Day of onset, median (min, max) | | 2.0 (1, 7) | 2.0 (1, 7) |
| Duration (days), median (min, max) | | 1.5 (1, 7) | 2.0 (1, 7) |
| Fatigue | |  |  |
| Any solicited adverse reactions | | 66 (44.0) | 68 (46.6) |
| Day of onset, median (min, max) | | 2.0 (1, 7) | 2.0 (1, 7) |
| Duration (days), median (min, max) | | 2.0 (1, 15) | 2.0 (1, 102) |
| Myalgia | |  |  |
| Any solicited adverse reactions | | 45 (30.0) | 51 (34.9) |
| Day of onset, median (min, max) | | 2.0 (1, 7) | 2.0 (1, 6) |
| Duration (days), median (min, max) | | 1.0 (1, 7) | 2.0 (1, 27) |
| Arthralgia | |  |  |
| Any solicited adverse reactions | | 31 (20.7) | 43 (29.5) |
| Day of onset, median (min, max) | | 2.0 (1, 7) | 2.0 (1, 7) |
| Duration (days), median (min, max) | | 1.0 (1, 7) | 1.0 (1, 7) |
| Nausea/Vomiting | |  |  |
| Any solicited adverse reactions | | 18 (12.0) | 23 (15.8) |
| Day of onset, median (min, max) | | 3.0 (1, 7) | 2.0 (1, 7) |
| Duration (days), median (min, max) | | 1.0 (1, 5) | 1.0 (1, 6) |
| Chills | |  |  |
| Any solicited adverse reactions | | 20 (13.3) | 31 (21.2) |
| Day of onset, median (min, max) | | 2.0 (1, 6) | 2.0 (1, 4) |
| Duration (days), median (min, max) | | 1.0 (1, 7) | 1.0 (1, 6) |
|  | |  |  |

Abbreviation: any, grade 1 or above.

N1 is the number of exposed participants who submitted any data for the event.

Percentages were based on the number of exposed participants who submitted any data for the event (N1).

Duration was calculated as the last day — the first day + 1 when the solicited adverse reaction was reported starting within the 7 days of injection.

If a participant reported events after both the first and second injections, the event selected for summarizing the day of onset and duration was the one with the higher toxicity grade. If the toxicity grades were the same, the event with the longer duration was selected. If the toxicity grades and durations were the same, the event with the earlier onset day was selected.

## Supplementary Table 3. Overall Summary of Unsolicited Adverse Events up to 28 Days After Any Injection (Safety Set)

|  | **mRNA-1345 50 µg (N = 150) n (%)** |
| --- | --- |
|  | |
| Unsolicited AEs up to 28 days after any injection,  regardless of relationship to study injection |  |
| All | 51 (34.0) |
| Serious | 9 (6.0) |
| Fatal | 0 |
| Reported as biopsy-proven organ rejection | 0 |
| Medically attended | 37 (24.7) |
| Leading to discontinuation from study injection | 0 |
| Leading to study discontinuation | 0 |
| Severe | 4 (2.7) |
| Nonserious^a^ | 42 (28.0) |
| Severe^a^ | 0 |
| At least 1 nonserious event^b^ | 46 (30.7) |
| Severe^b^ | 0 |
| Any AESI | 0 |
|  | |
| Unsolicited AEs up to 28 days after any injection, related to study injection |  |
| All | 3 (2.0) |
| Serious | 1 (0.7) |
| Fatal | 0 |
| Reported as biopsy-proven organ rejection | 0 |
| Medically attended | 1 (0.7) |
| Leading to discontinuation from study injection | 0 |
| Leading to study discontinuation | 0 |
| Severe | 1 (0.7) |
| Nonserious^a^ | 2 (1.3) |
| At least 1 nonserious event^b^ | 2 (1.3) |
| Any AESI | 0 |

Abbreviations: AE, adverse event; AESI, adverse event of special interest; AR, adverse reaction.

An AE was defined as any event not present before exposure to study injection or any event already present that worsened in intensity or frequency after exposure.

Severe AEs included both unsolicited severe AEs and grade ≥3 solicited ARs that met SAE criteria.

Percentages were based on the number of participants in the safety set.

^a^For each section, participants who did not report any serious AE were included in the summary of “nonserious AEs” and “severe/grade ≥3 nonserious AEs.”

^b^Participants with at least 1 nonserious AE were included.

## Supplementary Table 4. Overall Summary of Unsolicited Adverse Events up to DCO/EoS (Safety Set)

|  | **mRNA-1345 50 µg (N = 150) n (%)** |
| --- | --- |
|  | |
| Unsolicited AEs up to DCO/EoS, regardless of relationship to study injection |  |
| Serious | 43 (28.7) |
| Fatal | 1 (0.7) |
| Reported as biopsy-proven organ rejection | 5 (3.3) |
| Medically attended^a^ | 98 (65.3) |
| Severe medically attended^a^ | 35 (23.3) |
| Leading to discontinuation from study injection | 1 (0.7) |
| Leading to study discontinuation | 1 (0.7) |
| Severe | 35 (23.3) |
| Any AESI | 2 (1.3) |
| Any severe AESI | 1 (0.7) |
|  | |
| Unsolicited AEs up to DCO/EoS, related to study injection |  |
| Serious | 1 (0.7) |
| Fatal | 0 |
| Reported as biopsy-proven organ rejection | 0 |
| Medically attended^a^ | 1 (0.7) |
| Severe medically attended^a^ | 1 (0.7) |
| Leading to discontinuation from study injection | 0 |
| Leading to study discontinuation | 0 |
| Severe | 1 (0.7) |
| Any AESI | 0 |
| Any severe AESI | 0 |

Abbreviations: AE, adverse event; AESI, adverse event of special interest; AR, adverse reaction; DCO, data cutoff; EoS, end of study; SAE, serious adverse event.

An AE was defined as any event not present before exposure to study injection or any event already present that worsened in intensity or frequency after exposure.

Severe AEs included both unsolicited severe AEs and grade ≥3 solicited ARs that met SAE criteria.

Percentages were based on the number of participants in the safety set.

^a^Medically attended AEs were summarized up to Day 271/Month 9.

## Supplementary Table 5. Summary of Neutralizing Antibody Seroresponse Rate by Visit and Solid Organ Transplant Type (Per-Protocol Set)

|  | **mRNA-1345 50 µg** | | | |
| --- | --- | --- | --- | --- |
| **Timepoint**  **Statistic** | **Kidney Transplant (N = 47)** | **Liver Transplant  (N = 51)** | **Lung Transplant  (N = 45)** | **Total  (N = 143)** |
| **RSV-A neutralizing antibody titer** | |  |  |  |
| Day 29 |  |  |  |  |
| Seroresponse^a^ |  |  |  |  |
| n (%)^b^ | 21 (44.7) | 38 (74.5) | 14 (31.1) | 73 (51.0) |
| 95% CI^c^ | (30.2, 59.9) | (60.4, 85.7) | (18.2, 46.6) | (42.6, 59.5) |
|  |  |  |  |  |
| ≥2-fold increase from baseline^d^ |  |  |  |  |
| n (%)^b^ | 30 (63.8) | 42 (82.4) | 22 (48.9) | 94 (65.7) |
| 95% CI^c^ | (48.5, 77.3) | (69.1, 91.6) | (33.7, 64.2) | (57.3, 73.5) |
|  |  |  |  |  |
| Day 85 |  |  |  |  |
| Seroresponse^a^ |  |  |  |  |
| n (%)^b^ | 26 (56.5) | 40 (80.0) | 25 (55.6) | 91 (64.5) |
| 95% CI^c^ | (41.1, 71.1) | (66.3, 90.0) | (40.0, 70.4) | (56.0, 72.4) |
|  |  |  |  |  |
| ≥2-fold increase from baseline^d^ |  |  |  |  |
| n (%)^b^ | 40 (87.0) | 45 (90.0) | 35 (77.8) | 120 (85.1) |
| 95% CI^c^ | (73.7, 95.1) | (78.2, 96.7) | (62.9, 88.8) | (78.1, 90.5) |
|  |  |  |  |  |
| Day 181 |  |  |  |  |
| Seroresponse^a^ |  |  |  |  |
| n (%)^b^ | 22 (47.8) | 33 (66.0) | 18 (42.9) | 73 (52.9) |
| 95% CI^c^ | (32.9, 63.1) | (51.2, 78.8) | (27.7, 59.0) | (44.2, 61.4) |
|  |  |  |  |  |
| ≥2-fold increase from baseline^d^ |  |  |  |  |
| n (%)^b^ | 34 (73.9) | 40 (80.0) | 30 (71.4) | 104 (75.4) |
| 95% CI^c^ | (58.9, 85.7) | (66.3, 90.0) | (55.4, 84.3) | (67.3, 82.3) |
|  |  |  |  |  |
| **RSV-B neutralizing antibody titer** | |  |  |  |
| Day 29 |  |  |  |  |
| Seroresponse^a^ |  |  |  |  |
| n (%)^b^ | 19 (40.4) | 30 (58.8) | 8 (18.2) | 57 (40.1) |
| 95% CI^c^ | (26.4, 55.7) | (44.2, 72.4) | (8.2, 32.7) | (32.0, 48.7) |
|  |  |  |  |  |
| ≥2-fold increase from baseline^d^ |  |  |  |  |
| n (%)^b^ | 27 (57.4) | 41 (80.4) | 21 (47.7) | 89 (62.7) |
| 95% CI^c^ | (42.2, 71.7) | (66.9, 90.2) | (32.5, 63.3) | (54.2, 70.6) |
|  |  |  |  |  |
| Day 85 |  |  |  |  |
| Seroresponse^a^ |  |  |  |  |
| n (%)^b^ | 22 (46.8) | 38 (76.0) | 20 (44.4) | 80 (56.3) |
| 95% CI^c^ | (32.1, 61.9) | (61.8, 86.9) | (29.6, 60.0) | (47.8, 64.6) |
|  |  |  |  |  |
| ≥2-fold increase from baseline^d^ |  |  |  |  |
| n (%)^b^ | 35 (74.5) | 46 (92.0) | 32 (71.1) | 113 (79.6) |
| 95% CI^c^ | (59.7, 86.1) | (80.8, 97.8) | (55.7, 83.6) | (72.0, 85.9) |
|  |  |  |  |  |
| Day 181 |  |  |  |  |
| Seroresponse^a^ |  |  |  |  |
| n (%)^b^ | 20 (43.5) | 29 (58.0) | 14 (33.3) | 63 (45.7) |
| 95% CI^c^ | (28.9, 58.9) | (43.2, 71.8) | (19.6, 49.5) | (37.2, 54.3) |
|  |  |  |  |  |
| ≥2-fold increase from baseline^d^ |  |  |  |  |
| n (%)^b^ | 32 (69.6) | 44 (88.0) | 29 (69.0) | 105 (76.1) |
| 95% CI^c^ | (54.2, 82.3) | (75.7, 95.5) | (52.9, 82.4) | (68.1, 82.9) |

Abbreviations: CI, confidence interval; IRT, interactive response technology; LLOQ, lower limit of quantification; nAb, neutralizing antibody; RSV, respiratory syncytial virus; SOT, solid organ transplant; SRR, seroresponse rate; ULOQ, upper limit of quantification.

N1 is the number of participants with non-missing data at baseline and the corresponding post-baseline timepoint.

Antibody values reported as below the LLOQ were replaced by 0.5 × LLOQ. Values greater than the ULOQ were replaced by the ULOQ.

SOT type was derived from data collected via IRT.

^a^Seroresponse at a participant level was defined as a change from below the LLOQ to equal or above 4 × LLOQ, or at least a 4-fold increase if baseline was equal to or above the LLOQ.

^b^Number of participants who met the criterion at the timepoint. Percentages were based on N1.

^c^95% CI was calculated using the Clopper-Pearson method.

^d^≥z-fold increase from baseline at participant level was defined as a change from below the LLOQ to equal or above z × LLOQ, or at least a z-fold increase if baseline is equal to or above the LLOQ.

## Supplementary Table 6. Summary of Binding Antibody GMC and GMFR by Visit (Per-Protocol Set)

| **Timepoint**  **Statistic** | **Kidney Transplant**  **(N=47)** | **Liver**  **Transplant**  **(N=51)** | **Lung**  **Transplant**  **(N=45)** | **mRNA-1345 50 µg** **Total (N = 143)** |  |
| --- | --- | --- | --- | --- | --- |
| **RSV preF IgG antibody**  **(AU/mL)** |  |  |  |  |  |
| Baseline (Day 1) |  |  |  |  |  |
| n^a^ | 46 | 51 | 44 | 141 |  |
| GM concentration | 9448.87 | 11083.97 | 11308.30 | 10587.59 |  |
| 95% CI^b^ | (7455.48, 11975.24) | (8684.52, 14146.37) | (8406.92, 15211.00) | (9146.86, 12255.26) |  |
|  |  |  |  |  |  |
| Day 29 |  |  |  |  |  |
| n^a^ | 47 | 50 | 45 | 142 |  |
| GM concentration | 36408.26 | 80020.68 | 29135.53 | 44766.64 | |
| 95% CI^b^ | (25598.12, 51783.54) | (61355.33, 104364.36) | (20105.86, 42220.48) | (36681.38, 54634.04) | |
| N1 | 46 | 50 | 44 | 140 |  |
| GM fold rise | 3.71 | 7.19 | 2.56 | 4.18 |  |
| 95% CI^b^ | (2.88, 4.77) | (5.37, 9.63) | (1.88, 3.49) | (3.51, 4.99) |  |
|  |  |  |  |  |  |
| Day 85 |  |  |  |  |  |
| n^a^ | 46 | 50 | 44 | 140 |  |
| GM concentration | 54034.13 | 106781.16 | 47920.64 | 66363.89 |  |
| 95% CI^b^ | (38750.64, 75345.52) | (83887.07, 135923.40) | (33861.41, 67817.25) | (55294.75, 79648.90) |  |
| N1 | 45 | 50 | 43 | 138 |  |
| GM fold rise | 5.47 | 9.33 | 4.22 | 6.12 |  |
| 95% CI^b^ | (4.21, 7.10) | (7.21, 12.07) | (3.14, 5.67) | (5.20, 7.20) |  |
|  |  |  |  |  |  |
| Day 181 |  |  |  |  |  |
| n^a^ | 44 | 50 | 41 | 135 |  |
| GM concentration | 33255.01 | 51665.30 | 33793.34 | 39340.74 |  |
| 95% CI^b^ | (23798.45, 46469.23) | (39563.12, 67469.49) | (23774.48, 48034.27) | (32840.25, 47127.95) |  |
| N1 | 43 | 50 | 40 | 133 |  |
| GM fold rise | 3.35 | 4.80 | 2.92 | 3.68 |  |
| 95% CI^b^ | (2.58, 4.36) | (3.61, 6.39) | (2.15, 3.99) | (3.12, 4.34) |  |

Abbreviations: bAb, binding antibody; CI, confidence interval; GM, geometric mean; GMC, geometric mean concentration; GMFR, geometric mean fold rise; IRT, interactive response technology; LLOQ, lower limit of quantification; RSV, respiratory syncytial virus; ULOQ, upper limit of quantification.

N1 is the number of participants with non-missing data at baseline and the corresponding post-baseline timepoint.

Antibody values reported as below the LLOQ were replaced by 0.5 × LLOQ. Values greater than the ULOQ were replaced by the ULOQ.

^a^Number of participants with non-missing data at the timepoint (baseline or post-baseline).

^b^95% CI was calculated based on the *t* distribution of the log-transformed values or the difference in the log-transformed values for GM concentration and GM fold rise, respectively, then back-transformed to the original scale for presentation.

## Supplementary Table 7. Summary of Day 29, Day 85, and Day 181 Binding Antibody Seroresponse Rates (Per-Protocol Set)

| **Timepoint**  **Statistic** | **Kidney Transplant**  **(N=47)** | **Liver**  **Transplant**  **(N=51)** | **Lung**  **Transplant**  **(N=45)** | **mRNA-1345 50 µg Total (N = 143)** |
| --- | --- | --- | --- | --- |
| **RSV preF IgG antibody (AU/mL)** |  |  |  |  |
| Day 29 |  |  |  |  |
| Seroresponse^a^ |  |  |  |  |
| n (%)^b^ | 20 (43.5) | 37 (74.0) | 12 (27.3) | 69 (49.3) |
| 95% CI^c^ | (28.9, 58.9) | (59.7, 85.4) | (15.0, 42.8) | (40.7, 57.9) |
|  |  |  |  |  |
| ≥2-fold increase from baseline^d^ |  |  |  |  |
| n (%)^b^ | 33 (71.7) | 42 (84.0) | 22 (50.0) | 97 (69.3) |
| 95% CI^c^ | (56.5, 84.0) | (70.9, 92.8) | (34.6, 65.4) | (60.9, 76.8) |
|  |  |  |  |  |
| Day 85 |  |  |  |  |
| Seroresponse^a^ |  |  |  |  |
| n (%)^b^ | 32 (71.1) | 43 (86.0) | 20 (46.5) | 95 (68.8) |
| 95% CI^c^ | (55.7, 83.6) | (73.3, 94.2) | (31.2, 62.3) | (60.4, 76.4) |
|  |  |  |  |  |
| ≥2-fold increase from baseline^d^ |  |  |  |  |
| n (%)^b^ | 38 (84.4) | 48 (96.0) | 33 (76.7) | 119 (86.2) |
| 95% CI^c^ | (70.5, 93.5) | (86.3, 99.5) | (61.4, 88.2) | (79.3, 91.5) |
|  |  |  |  |  |
| Day 181 |  |  |  |  |
| Seroresponse^a^ |  |  |  |  |
| n (%)^b^ | 19 (44.2) | 28 (56.0) | 15 (37.5) | 62 (46.6) |
| 95% CI^c^ | (29.1, 60.1) | (41.3, 70.0) | (22.7, 54.2) | (37.9, 55.5) |
|  |  |  |  |  |
| ≥2-fold increase from baseline^d^ |  |  |  |  |
| n (%)^b^ | 30 (69.8) | 41 (82.0) | 27 (67.5) | 98 (73.7) |
| 95% CI^c^ | (53.9, 82.8) | (68.6, 91.4) | (50.9, 81.4) | (65.3, 80.9) |

Abbreviations: bAb, binding antibody; CI, confidence interval; IRT, interactive response technology; LLOQ, lower limit of quantification; RSV, respiratory syncytial virus; SRR, seroresponse rate; ULOQ, upper limit of quantification.

N1 is the number of participants with non-missing data at baseline and the corresponding post-baseline timepoint.

Antibody values reported as below the LLOQ were replaced by 0.5 × LLOQ. Values greater than the ULOQ were replaced by the ULOQ.

^a^Seroresponse at a participant level was defined as a change from below the LLOQ to equal or above 4 × LLOQ, or at least a 4-fold increase if baseline was equal to or above the LLOQ.

^b^Number of participants who met the criterion at the timepoint. Percentages were based on N1.

^c^95% CI was calculated using the Clopper-Pearson method.

^d^≥z-fold increase from baseline at participant level was defined as a change from below the LLOQ to equal or above z × LLOQ, or at least a z-fold increase if baseline was equal to or above the LLOQ.
